# Hypoxia-Induced Reactivity of Tumor-Associated Astrocytes Affects Glioma Cell Properties

**DOI:** 10.3390/cells10030613

**Published:** 2021-03-10

**Authors:** Vasiliki Pantazopoulou, Pauline Jeannot, Rebecca Rosberg, Tracy J. Berg, Alexander Pietras

**Affiliations:** Division of Translational Cancer Research, Department of Laboratory Medicine, Lund University, 223 63 Lund, Sweden; vasiliki.pantazopoulou@med.lu.se (V.P.); pauline.jeannot@med.lu.se (P.J.); rebecca.rosberg@med.lu.se (R.R.); tracy.berg@med.lu.se (T.J.B.)

**Keywords:** astrocytes, glioma microenvironment, tumor hypoxia

## Abstract

Glioblastoma is characterized by extensive necrotic areas with surrounding hypoxia. The cancer cell response to hypoxia in these areas is well-described; it involves a metabolic shift and an increase in stem cell-like characteristics. Less is known about the hypoxic response of tumor-associated astrocytes, a major component of the glioma tumor microenvironment. Here, we used primary human astrocytes and a genetically engineered glioma mouse model to investigate the response of this stromal cell type to hypoxia. We found that astrocytes became reactive in response to intermediate and severe hypoxia, similarly to irradiated and temozolomide-treated astrocytes. Hypoxic astrocytes displayed a potent hypoxia response that appeared to be driven primarily by hypoxia-inducible factor 2-alpha (HIF-2α). This response involved the activation of classical HIF target genes and the increased production of hypoxia-associated cytokines such as TGF-β1, IL-3, angiogenin, VEGF-A, and IL-1 alpha. In vivo, astrocytes were present in proximity to perinecrotic areas surrounding HIF-2α expressing cells, suggesting that hypoxic astrocytes contribute to the glioma microenvironment. Extracellular matrix derived from hypoxic astrocytes increased the proliferation and drug efflux capability of glioma cells. Together, our findings suggest that hypoxic astrocytes are implicated in tumor growth and potentially stemness maintenance by remodeling the tumor microenvironment.

## 1. Introduction

Glioblastoma (GBM) is the most common high-grade glioma and represents one of the most aggressive tumor types, with a dismal five-year survival [1]. Despite treatment with surgery, irradiation, and chemotherapy, all patients recur [2]. Treatment resistance, and the ensuing tumor recurrence, has been associated with the presence of tumor cells with stem-like cell properties [3] that are located in different tumor areas such as the hypoxic niche [4].

Hypoxia is a hallmark of glioblastoma lesions [5]. It is regulated mainly by two transcription factors, hypoxia-inducible factor 1-alpha and 2-alpha (HIF-1α and HIF-2α) [6], along with the less studied HIF-3α [7]. The HIF factors are regulated in an oxygen-dependent manner by prolyl hydroxylases and an asparagine hydroxylase [8] or in an oxygen-independent manner by various growth factors or the hypoxia-associated factor, amongst other mechanisms [9]. Hypoxia has been associated with an increase in glioma stem-like cell properties [4,10,11], glioma growth [12], and treatment resistance [13,14], as well as changes in cancer cell metabolism and pH [15,16]. Our lab and others have shown that differential stabilization of HIF factors in glioma stem-like cells maintains the stemness phenotype of these cells [5,17,18,19,20,21]. Hypoxic niches contain not only tumor cells, but also other cell types in the tumor microenvironment [5,22] that are also affected by low oxygen tension.

Astrocytes are one of the most abundant stromal cell types in the GBM microenvironment [22]. They have been implicated in GBM growth and invasion [23,24,25,26,27], they activate chemotherapy resistance programs in cancer cells, and interact with immune cells in brain tumors [28,29,30,31]. Astrocytes respond to brain injury by becoming reactive and entering a process termed reactive astrogliosis [32]. Recently, our lab showed that astrocytes exposed to radiation become reactive and subsequently promote GBM stemness, potentially contributing to future recurrence [33]. However, the effect of other microenvironmental stresses on astrocytes, such as hypoxia, remains elusive in the context of brain tumors.

Here, we used primary human astrocytes and a genetically engineered glioma mouse model to investigate the response of this stromal cell type to hypoxia. We found that astrocytes become reactive in response to intermediate and severe hypoxia, and that extracellular matrix produced by hypoxic astrocytes increased proliferation and drug efflux capabilities of glioma cells. Together, our findings suggest that hypoxic astrocytes adopt a tumor-supportive role in gliomas.

## 2. Materials and Methods

### 2.1. Generation of Murine Gliomas

Gliomas were generated in Nestin-tv-a (Ntv-a) mice by intracranially injecting the chicken fibroblast DF-1 cells (ATCC ^®^ CRL-12203^™^, ATCC, Manassas, VA, USA) expressing replication-competent avian sarcoma-leukosis virus long-terminal repeat with slice acceptor (RCAS) encoding human platelet-derived growth factor B (PDGFB) and RCAS-short hairpin p53 (shp53) into the neonatal brain, as previously described [34,35]. Mice were sacrificed upon development of glioma symptoms. All animal procedures were performed in accordance with the European Union directive about animal rights and approved by the Lund ethical committee (M-16123/19).

### 2.2. Cell Culture and Treatments

Primary human astrocytes (3H Biomedical, Uppsala, Sweden) were cultured in astrocyte medium (3H Biomedical) and passaged using Accutase (Thermo Fisher Scientific, Waltham, MA, USA). Experiments were performed using 3 independent astrocyte lines, corresponding to 3 individual donors. Cells were cultured at 37 °C in a humidified incubator containing 5% CO_2_ and 21% O_2_, until placed in other oxygen tensions. Physoxia and hypoxia were generated in a Whitney H35 Hypoxystation (Don Whitley Scientific, Bingley, United Kingdom) (1% O_2_) or an InvivO_2_ 400 Hypoxia Workstation (Baker Ruskinn, Bridgend, United Kingdom) (5% or 0.1% O_2_). Cells were treated with a single dose of 10 Gy in a CellRad x-ray irradiator (Faxitron), with 200 μM temozolomide (Sigma-Aldrich, St. Louis, MO, USA) or an equivalent volume of Dimethyl sulfoxide (DMSO) (Sigma-Aldrich).

PDGFB-induced glioma primary cultures (PIGPC) were isolated as previously described [36]. U251MG cells were obtained from ATCC. PIGPC and U251MG cells were cultured in Dulbecco’s Modified Eagle’s medium (DMEM) (Corning, New York, NY, USA) supplemented with 10% fetal bovine serum (FBS) (Biological Industries, Beit HaEmek, Israel) and 1% pen–strep (Corning). Cells were cultured at 37 °C in a humidified incubator containing 5% CO_2_ and 21% O_2_.

### 2.3. Immunofluorescence

Whole brains were embedded in Optimal cutting temperature compound (OCT) (Thermo Fisher Scientific) and frozen in ice-cold isopentane. Sections were air-dried for 30 min, fixed in ice-cold acetone, and permeabilized in 0.3% Triton X-100 (Sigma-Aldrich) in phosphate-buffered saline (PBS). Cells were fixed in 4% paraformaldehyde (PFA) and permeabilized in 0.3% Triton X-100 (Sigma-Aldrich) in PBS followed by blocking with 1% bovine serum albumin (BSA) in PBS. Sections or cells were then incubated overnight at 4 °C with the following antibodies in 1% BSA: Glial fibrillary acidic protein (GFAP) (13-03300, 1:1500 dilution, Thermo Fisher Scientific), vimentin (ab45939, 1:500 dilution, Abcam, Cambridge, United Kingdom), phalloidin (U0281, 1:500 dilution, Abnova, Taipei, Taiwan), and HIF-2α (ab199, 1:100 dilution, Abcam). Sections stained for HIF-2α were incubated for 30 min with rabbit linker (DAKO, Agilent Technologies, Santa Clara, CA, USA). Sections were incubated with the appropriate Alexafluor-conjugated secondary antibodies (Invitrogen, Carlsbad, CA, USA) in the presence of 4′,6-diamidino-2-phenylindole (DAPI) (Sigma-Aldrich) or Hoechst 33,342 (Sigma-Aldrich) for 1 h. Images were captured using an Olympus BX63 microscope, a DP80 camera, and cell-Sens Dimension v 1.12 software (Olympus Corporation, Tokyo, Japan). Cell area, cell perimeter, and vimentin fluorescence intensity were quantified for each cell using CellProfiler 4.0.6 [37].

### 2.4. Western Blot

Whole cell lysates were prepared in radioimmunoprecipitation assay (RIPA) buffer supplemented with a complete protease inhibitor cocktail (Roche Life Science, Penzberg, Germany). Equal amounts of sample (80-100 μg protein) were diluted in Laemmli buffer (Bio-Rad Laboratories, Hercules, CA, USA) with dithiothreitol (DTT) and were boiled for 5 min. Lysates were separated on 7.5% Mini-PROTEAN^®^ TGX™ Precast Protein Gels (Bio-Rad Laboratories) and proteins were transferred to polyvinylidene fluoride (PVDF) membranes using a Transblot Turbo System (Bio-Rad Laboratories). Membranes were blocked in 5% non-fat dry milk/Tris buffered saline with Tween 20 (TBS-T), and incubated overnight with the following primary antibodies: HIF-1α (NB100-479, 1:500 dilution, Novus Biologicals, Centennial, CO, USA), HIF-1α (610958, 1:500 dilution, BD Bioscience, San Jose, CA; USA), HIF-2α (ab199, 1:500 dilution, Abcam), Succinate Dehydrogenase Complex Flavoprotein Subunit A (SDHA) (ab14715, 1:4000 dilution, Abcam). After washing, membranes were incubated for 1 h with secondary antibodies (1:5000 dilution, Jackson ImmunoResearch, West Grove, PA, USA). Images were acquired using a LAS-3000 Imager (Fujifilm, Tokyo, Japan). Band intensity was quantified using Fiji [38].

### 2.5. Real-Time qPCR

RNA was isolated using the RNeasy Mini Kit together with the Qiashredder Kit (Qiagen, Hilden, Germany) according to manufacturer’s instructions, and cDNA was synthesized using random primers and the Multi-Scribe reverse transcriptase enzyme (Applied Biosystems, Foster City, CA, USA). The amplifications were run using a QuantStudio 7 real-time PCR system (Applied Biosystems) with SYBR® Green Master Mix (Applied Biosystems). Relative gene expression was normalized to the expression of three housekeeping genes (*UBC, SDHA,* and *YWHAZ*) using the comparative Ct method [39]. Primers used in the study are provided in Table 1.

### 2.6. Cytokine Array

The Human Cytokine Antibody Array (ab133997, Abcam) was used according to manufacturer’s instructions on 200 μg of lysates from astrocytes derived from 3 individual donors after culture for 72 h in 21%, 1%, or 0.1% O_2_.

### 2.7. Astrocyte-Derived Matrix

Confluent astrocytes were cultured on 0.2% gelatin for 10 days in astrocyte medium supplemented with 50 μg/mL L-ascorbic acid (Sigma-Aldrich) in different oxygen tensions. Cells were decellularized in 0.4 mM NH_4_OH (Honeywell, Charlotte, NC, USA), 0.5% Triton X-100 in PBS with 1 mM CaCl_2_, and 0.5 mM MgCl_2_ (PBS-MC) at 37 °C, washed with PBS-MC, then treated with 10 μg/mL DNAse I (Roche Life Science) for 1 h at 37 °C. The matrix was stored in PBS-MC at 4 °C until use. Astrocyte-derived matrix (ADM) was generated by astrocytes cultured in normoxia [(21% O_2_, ADM_21_), intermediate hypoxia (1% O_2_, ADM_1_), or severe hypoxia (0.1% O_2_, ADM_0.1_).

### 2.8. Colony Assay

U251MG or PIGPC cells were plated at 200 or 150 cells/well, respectively, in a 6-well dish on ADM_21_ or ADM_1_ in DMEM. Cells were cultured for 2 weeks or until visible colonies formed. Colonies were fixed in 4% PFA, stained with 0.01% crystal violet for 1 h, photographed on a LAS-3000 Imager (Fujifilm), then counted manually (PIGPC colonies) or using Fiji [38] followed by visual confirmation (U251MG colonies).

### 2.9. Side Population

The side population assay was performed as previously described [40]. Briefly, cells were resuspended at 1 × 10^6^ cells/mL and incubated at 37 °C for 30 min with or without fumitremorgin (FTC) (Sigma-Aldrich). Then, cells were incubated for an additional 90 min with 5 mg/mL Hoechst 33342 (Sigma-Aldrich) with periodic shaking. Cells were analyzed on a FACSVerse instrument (BD Bioscience) equipped with a 405-nm violet laser. Dual wavelength detection was performed using 448/45 (Hoechst 33342-blue) and 613/18 (Hoechst 33342-red) filters. Data were analyzed using FlowJo vs. 10.7.1 (BD Bioscience).

### 2.10. Statistical Analysis

All statistical analysis was performed in GraphPad Prism vs. 8.1.2 or R 4.0.0. Statistical tests and number of replicates are indicated in figure legends. All t-tests were two-tailed. Significance is expressed as *p* values (* *p* < 0.05, *** *p* < 0.001).

## 3. Results

### 3.1. Astrocytes Adopt a Reactive Phenotype in Response to Stress Related to the Glioma Microenvironment

**Figure 1:**
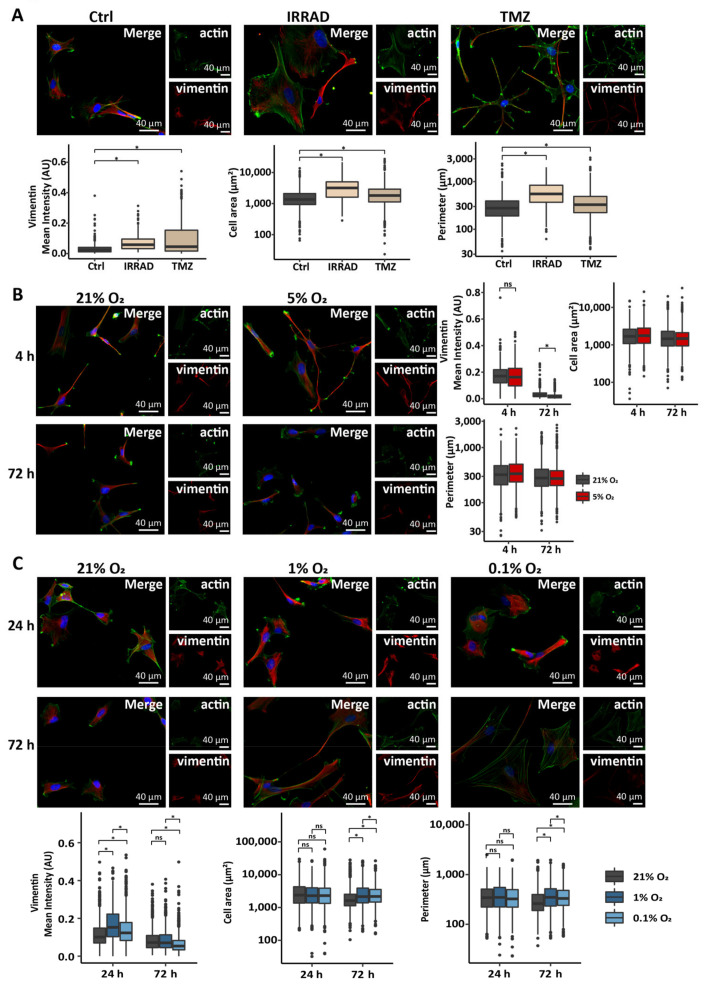
Astrocytes adopt a reactive phenotype in response to stress related to the glioma microenvironment. (**A**) Representative images and quantification of vimentin fluorescence intensity, cell area, and cell perimeter in astrocytes after 72 h in culture after a single dose of 10 Gy (IRRAD) or treatment with 200 μM temozolomide (TMZ) or DMSO (Ctrl). (**B**) Representative images and quantification of vimentin fluorescence intensity, cell area, and cell perimeter in astrocytes after 4 h or 72 h in culture at 21% or 5% O_2_. (**C**) Representative images and quantification of vimentin fluorescence intensity, cell area, and cell perimeter in astrocytes after 24 h or 72 h in culture at 21%, 1% or 0.1% O_2_. AU, arbitrary unit. Data represent one replicate from two (A) or three (B, C) independent astrocyte lines. * *p* ≤ 0.05; ns, not significant; one-way ANOVA or Welch’s ANOVA (post-hoc test: pairwise t-test).

Astrocytes respond to a variety of damages to the brain by becoming reactive, a process that leads to the upregulation of vimentin expression and changes in cell morphology [32,41]. These damages include extrinsic factors, such as radiation and chemotherapy treatment, or could also present as intrinsic factors of the tumor microenvironment, such as hypoxia. As we have previously reported [33], irradiation induced reactive astrogliosis in primary astrocyte cultures in vitro and astrocytes exposed to a single dose of 10 Gy exhibited elevated levels of vimentin (Figure 1A) as well as somatic hypertrophy, shown by an increase in both cell area and cell perimeter (Figure 1A). Interestingly, astrocytes treated with temozolomide, a chemotherapeutic agent frequently administered after or while patients undergo radiation treatment [1], also showed an increase in features of reactive astrogliosis (Figure 1A), similar to that observed after irradiation.

To determine the response of astrocytes to an intrinsic stressor of the tumor microenvironment, such as hypoxia, we maintained astrocytes in culture under normoxic (21% O_2_), physoxic (5% O_2_), intermediate hypoxic (1% O_2_), and severe hypoxic (0.1% O_2_) conditions [42]. Short- or long-term culture of astrocytes in physoxia did not lead to an increase in vimentin expression or changes in morphology (Figure 1B), further supporting that 5% O_2_ closely resembles physiological O_2_ tension in the brain [42,43]. Interestingly, astrocytes exposed to intermediate or severe hypoxia showed an increase in markers of reactive astrogliosis. Namely, astrocytes expressed elevated levels of vimentin after 24 h in intermediate or severe hypoxia (Figure 1C), followed by an increase in cell area and cell perimeter persistent after 72 h in culture in hypoxic conditions (Figure 1C). The observed increase in vimentin expression levels in astrocytes exposed to hypoxia for 24 h reverted to the levels of the normoxic control or even below that after 72 h in culture (Figure 1C). This indicated that hypoxia induces the various features of reactive astrogliosis to a different extent and some of these features can be reverted. These data support that astrocytes respond to extrinsic (radiation or temozolomide treatment) or intrinsic (intermediate or severe hypoxia) stimuli, initiated either during treatment or tumor growth, by adopting a reactive phenotype.

### 3.2. Low Oxygen Tension Induces a Strong Hypoxic Response in Astrocytes

**Figure 2:**
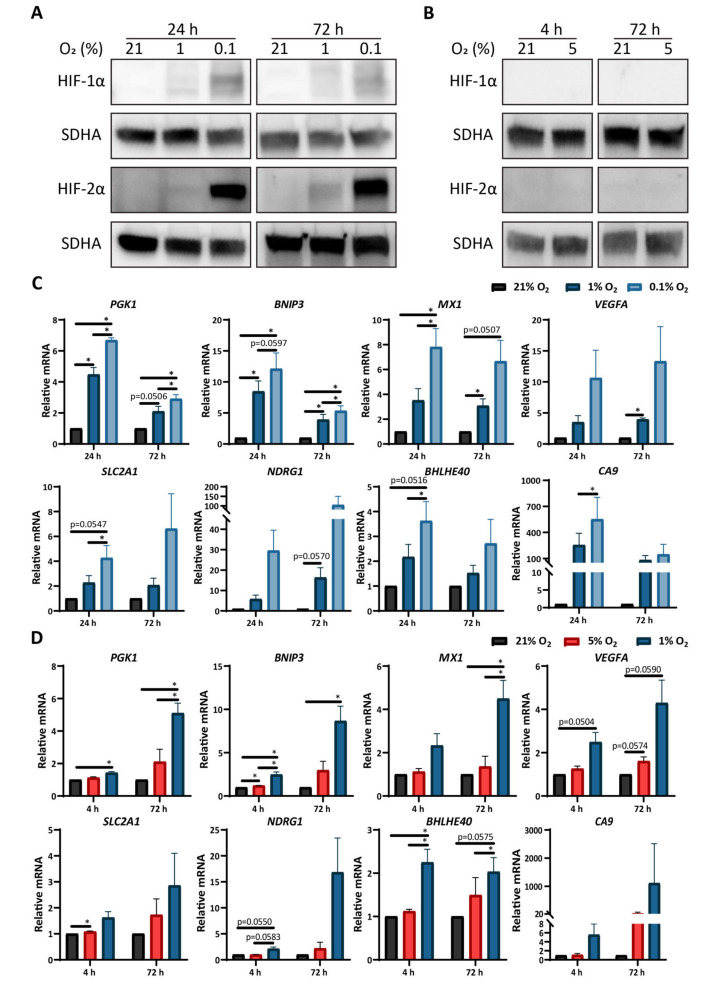
Low oxygen tension induces a strong hypoxic response in astrocytes. (**A**) Representative images of Western blots showing HIF-1α, HIF-2α, and SDHA (loading control) expression in astrocytes cultured for 24 or 72 h at 21%, 1%, or 0.1% O_2_. (**B**) Representative images of Western blots showing HIF-1α and HIF-2α expression in astrocytes cultured for 4 or 72 h at 21% or 5% O_2_. (**C**) qPCR data for relative mRNA expression of *PGK1*, *BNIP3*, *MX1*, *VEGFA*, *SLC2A1*, *NDRG1*, *BHLHE40*, and *CA9* in astrocytes cultured for 24 or 72 h at 21%, 1%, or 0.1% O_2_. * *p* ≤ 0.05 vs. normoxic controls, one-way ANOVA (Tukey’s multiple comparisons test). (**D**) qPCR data for relative mRNA expression of *PGK1*, *BNIP3*, *MX1*, *VEGFA*, *SLC2A1*, *NDRG1*, *BHLHE40*, and *CA9* in astrocytes cultured for 4 or 72 h at 21%, 5% O_2,_ or 1% O_2_. * *p* ≤ 0.05 vs. normoxic controls, one-way ANOVA (Tukey’s multiple comparisons test). All data represent mean ± SEM. All data represent one replicate from three independent astrocyte lines.

To further characterize how primary astrocytes respond to hypoxia, we performed Western blot analysis of HIF-1α and HIF-2α, the two transcription factors that largely orchestrate cellular responses to hypoxia [6]. The culture of astrocytes in intermediate (1% O_2_) or severe (0.1% O_2_) hypoxia led to changes in the levels of HIF transcription factors. More specifically, HIF proteins were not detectable in normoxic astrocytes, while cells exposed to intermediate or severe hypoxia for either 24 or 72 h showed an upregulation of both HIF-1α and HIF-2α (Figure 2A). Astrocytes exposed to severe hypoxia exhibited an increased accumulation of HIF proteins compared to cells exposed to intermediate hypoxia at both time points (Figure S1A). The culture of primary astrocytes in physoxia (5% O_2_) did not alter the protein levels of either transcription factor compared to cells cultured in normoxia (Figure 2B). Notably, astrocytes cultured under severe hypoxia for 72 h exhibited higher stabilization of HIF-2α compared to HIF-1α relative to astrocytes cultured in intermediate hypoxia. In contrast, human glioma cells cultured under the same conditions stabilized both transcription factors to similar degrees (Figure S1A).

We next performed RT-qPCR to evaluate the expression levels of a panel of classical HIF target genes. Astrocytes exposed to intermediate hypoxia showed an induction of four genes (*PGK1*, *BNIP3*, *MX1*, *VEGFA*) either at both timepoints or only after 72 h in culture (Figure 2C), as well as a strong but non-significant induction of two genes (*NDRG1*, *CA9*). Similarly, astrocytes exposed to severe hypoxia showed the induction of six genes (*PGK1*, *BNIP3, MXI1, SLC2A1*, *BHLHE40*, *CA9*) at one or both time points, as well as a strong but non-significant induction of two genes (*VEGFA*, *NDRG1*) (Figure 2C). In line with the HIF expression data, the mRNA levels of most of the classical HIF target genes were higher in astrocytes exposed to severe hypoxia compared to those exposed to intermediate hypoxia (Figure 2C). As expected by the lack of HIF-1α or HIF-2α protein stabilization, astrocytes exposed to physoxia showed no or minimal induction of hypoxia-associated genes after either 4 or 72 h in culture (Figure 2D). Interestingly, astrocytes exposed to intermediate hypoxia for 4 h showed a significant upregulation of HIF target genes compared to either 21% or 5% O_2_ cultured astrocytes (Figure 2D), but to lower levels compared to astrocytes exposed to intermediate hypoxia for 24 h (Figure 2C, D). Together, these data show that the response of astrocytes to hypoxia is highly regulated by HIF factors. Furthermore, they suggest that the response of the astrocytes to severe hypoxia might be preferentially regulated by HIF-2α and that astrocytes require severe hypoxia to fully engage the hypoxic response machinery.

### 3.3. Astrocytes Produce Hypoxia-Related Cytokines In Vitro and Are Present in Hypoxic Areas In Vivo

**Figure 3:**
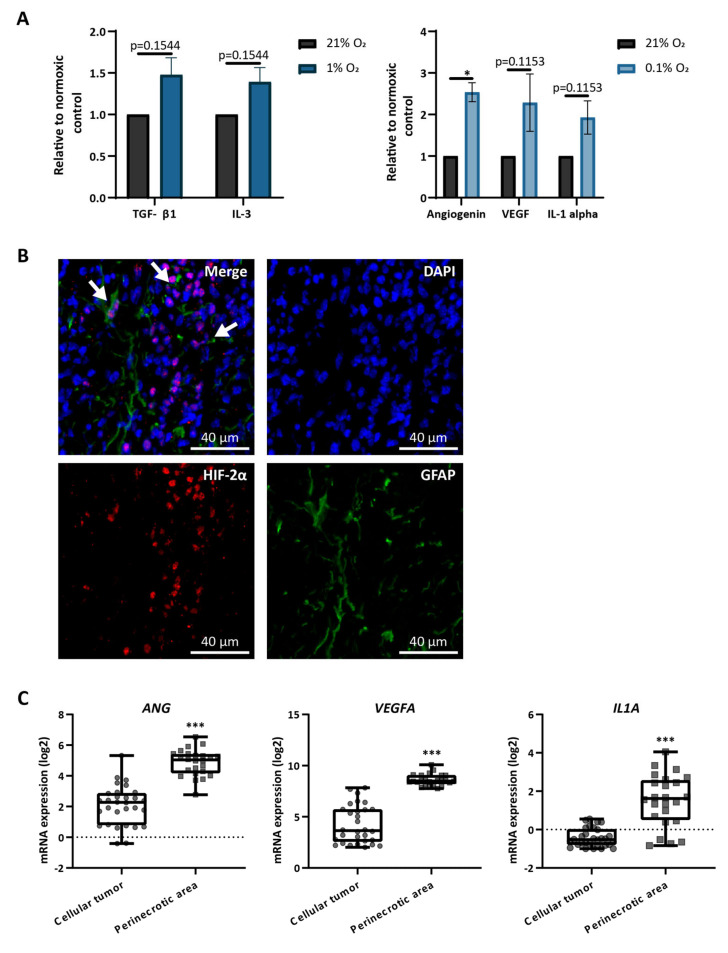
Astrocytes produce hypoxia-related cytokines in vitro and are present in hypoxic areas in vivo. (**A**) Quantification of cytokine arrays in lysates from three independent astrocyte lines cultured for 72 h at 21%, 1%, or 0.1% O_2_. Bars represent the mean change of each protein relative to the levels of the normoxic control. * *p* < 0.05 vs. normoxic controls, multiple t-tests (Holm–Sidak multiple comparisons test). Data represent mean ± SEM. (**B**) Representative image of immunofluorescent staining showing GFAP and HIF-2α expression in hypoxic areas of a PDGFB- and shp53-induced murine glioma. White arrows indicate locations of proximity of astrocytes (GFAP positive) to hypoxic cells (HIF-2α positive). A total of 5 hypoxic regions per tumor from 4 independent tumors were analyzed. (**C**) *ANG*, *VEGFA*, and *IL1A* mRNA expression in the indicated histological region of gliomas. *** *p* < 0.001, two-sided unpaired t-test.

To screen for changes in protein production in hypoxic astrocytes, we performed a cytokine array in three independent primary astrocyte lines cultured under normoxia, intermediate hypoxia, or severe hypoxia. Despite an expected variability in the identified targets (Table S1), all three lines showed an upregulation of TGF-β1 and IL-3 in response to culture in intermediate hypoxia and an upregulation of angiogenin, VEGF-A, and IL-1 alpha in response to culture in severe hypoxia (Figure 3A). All of these cytokines have been previously associated with increased tumor growth [44,45,46,47,48].

To examine the presence of astrocytes in hypoxic areas in vivo, we stained whole brain sections from glioma-bearing mice for HIF-2α (to mark the hypoxic areas) and GFAP (to mark the activated astrocytes) (Figure 3B). Astrocytes were found in proximity to perinecrotic areas, surrounding HIF-2α positive cells (Figure 3B). Analysis of the Ivy Glioblastoma Atlas Project [49] showed that the three cytokines produced by astrocytes in severe hypoxia are highly expressed in perinecrotic areas in human GBM (Figure 3C). Considering the presence of astrocytes in perinecrotic areas (Figure 3B), this could indicate that astrocytes are one source of these cytokines in the tumor microenvironment. These data suggest that astrocytes alter their proteome and might be altering the hypoxic tumor microenvironment.

### 3.4. Extracellular Matrix from Hypoxic Astrocytes Alters the Properties of Glioma Cells

Considering the changes we observed in hypoxia-treated astrocytes, both in morphology but also in gene and protein expression levels, we examined whether hypoxic astrocytes alter properties of glioma cells.

**Figure 4:**
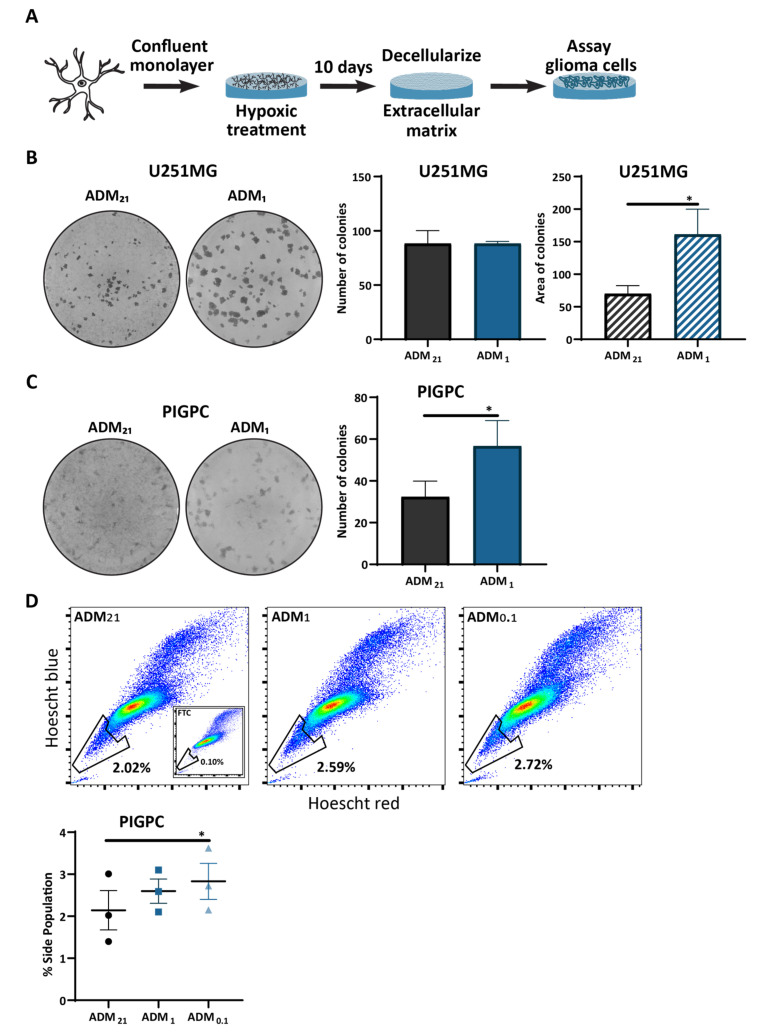
Extracellular matrix from hypoxic astrocytes alters the properties of glioma cells. (**A**) Experimental design. (**B**) Representative images of colony forming ability of U251MG glioma cells plated on clonal density on ADM_21_ or ADM_1_ in triplicate wells and quantification of colony number and size. * *p* < 0.05, ratio paired t-test. (**C**) Representative images of the colony forming ability of PIGPC glioma cells plated on clonal density on ADM_21_ or ADM_1_ in triplicate wells and quantification of colony number. * *p* < 0.05, ratio paired t-test. (**D**) Side population assay of PIGPC cells cultured on ADM_21_, ADM_1_, or ADM_0.1_. The graph shows quantification across three independent experiments. * *p* < 0.05, one-way ANOVA (Tukey’s multiple comparisons test). All data represent mean ± SEM. All data represent one replicate from three independent astrocyte lines.

We recently identified cell-derived matrix from astrocytes activated by irradiation as a promoter of glioma cell stemness [33]. Because we found astrocytes throughout the tumor (Figure 3B), we set out to examine the effect of hypoxic astrocytes on glioma cell growth by purifying extracellular matrix generated by astrocytes cultured in normoxia (ADM_21_) and intermediate hypoxia (ADM_1_) and determining the effect of ADM on the colony forming ability of glioma cells growing on it (Figure 4A). Human glioma U251MG cells showed no difference in the number of colonies they formed when cultured on clonal densities on matrix from the differentially treated astrocytes, but individual colonies formed on ADM_1_ had a bigger area compared to colonies on ADM_21_ (Figure 4B). Moreover, PDGFB-induced glioma primary cells (PIGPC) derived from RCAS-PDGFB-induced gliomas in Nestin-tv-a Ink4a/Arf-/- mice showed increased colony numbers, as well as visibly larger colonies when cultured on ADM_1_ compared to colonies formed on ADM_21_ (Figure 4C). These data suggest that astrocyte matrix produced under hypoxia can promote glioma cell proliferation.

Because we found astrocytes in proximity to hypoxic cells (Figure 3B), and these areas are a niche for glioma stem-like cells [5,17,18], we examined the effect of ADM on glioma cell drug efflux capacity, as measured by the side population (SP) assay, one measure of cell stemness [40,50]. PIGPC were cultured on ADM from astrocytes cultured in normoxia, intermediate hypoxia, and severe hypoxia, and we measured the SP of the cells (Figure S1B). PIGPC cells cultured on ADM_0.1_ showed an increase in SP compared to cells cultured on either ADM_21_ or ADM_1_ (Figure 4D). This suggests that matrix from hypoxic astrocytes, similarly to that from irradiated astrocytes [33], can promote drug efflux by glioma cells.

## 4. Discussion

Glioblastoma is characterized by extensive areas of hypoxia where both tumor cells and stromal cells experience low oxygen tensions [5]. Hypoxia affects the phenotype of glioma cells and it is becoming clearer that it also alters the stromal cells that surround perinecrotic areas [51,52]. Exactly how astrocytes, a big compartment of the GBM stroma, respond to hypoxia and how hypoxic astrocytes affect glioma cells is not fully understood. In ischemic injuries, which are also characterized by a decrease in oxygen tension, it has been established that astrocytes become reactive, alter their function, and adopt a neuroprotective role [53,54]. In this study, we show that hypoxia induces a reactive phenotype in astrocytes, similar to the reactivity that astrocytes show in response to extrinsic stresses of the tumor microenvironment, such as radiation treatment and chemotherapy. We found that hypoxic astrocytes alter their morphology and vimentin expression, and that TGF-β1 and IL-1 alpha expression are upregulated in astrocytes exposed to intermediate and severe hypoxia, respectively. These factors are well-established regulators of reactive astrogliosis [41] and their expression by hypoxic astrocytes supports our conclusion that astrocytes become reactive in the presence of low oxygen.

Astrocytes exposed to intermediate and severe hypoxia increased the stabilization of HIF factors as well as the expression of several hypoxia-regulated genes. Protein levels of both HIF-1α and HIF-2α were higher in astrocytes exposed to severe compared to intermediate hypoxia, with astrocytes exhibiting a higher stabilization of HIF-2α compared to HIF-1α when cultured in severe hypoxia for 72 h. The preferential stabilization of HIF-2α after 72 h in hypoxia supports previous reports on differential HIF stabilization over time [55,56]. The stabilization of the HIFs in astrocytes under hypoxic conditions is further supported by the higher mRNA expression of several HIF-regulated genes in cells cultured under severe compared to intermediate hypoxic conditions, indicating that astrocytes require very low oxygen tensions to fully deploy the hypoxia response pathway. These findings collectively suggest that since astrocytes cultured at both intermediate and severe hypoxia showed morphological and molecular changes, astrocytes alter their phenotype in response to low oxygen regardless to the extent of hypoxia. Interestingly, some features of reactivity, such as vimentin expression, seem to be more transient and revert to baseline levels, while others, such as increased somatic hypertrophy, last longer.

In this study, we refer to normoxic cells as cells cultured in 21% O_2_. This oxygen tension has been historically used to represent normal oxygen levels in cultures but is far from the physiological oxygen tension in the brain, which is 3–4% O_2_ on average. [42]. Here, we used 5% O_2_ to represent the physiological oxygen tension of the brain (physoxia). Astrocytes cultured in physoxia did not show any of the hallmarks of reactivity, or of a response to hypoxia. This might indicate that astrocytes are more tolerant to the high oxygen tension, relative to physiological oxygen levels in the brain, of normoxic cultures [42] compared to other cell types.

Reactive astrocytes have been associated with several aspects of glioma formation and tumor growth [57]. Microarray gene expression analysis of hypoxic astrocytes has shown that astrocytes upregulate several genes associated with angiogenesis, including *ANG* and *VEGF*, in response to hypoxia [58]. In our study, angiogenin, the protein encoded by *ANG*, was produced by astrocytes cultured under severe hypoxic conditions. Angiogenin has been implicated in tumor cell proliferation [47,59] and was found to interact with extracellular matrix proteins, such as fibulin-1 [60]. In a recent study by our lab, we detected fibulin-1 in matrix produced by astrocytes [33]. An interaction between angiogenin and fibulin-1 could provide one possible mechanism behind the increased proliferation we observed when glioma cells were grown on matrix generated by hypoxic astrocytes. Here, we also show that astrocytes exposed to hypoxia upregulate the gene and protein expression levels of VEGF-A, which, once secreted to the microenvironment, can increase the invasion of glioma cells by activating C-X-C chemokine receptor type 4 (CXCR4) signaling [61,62]. Our observation that astrocytes exposed to hypoxia upregulate the expression of VEGF-A can explain previous findings of increased invasion and proliferation of glioma cells grown in the presence of medium from hypoxic astrocytes [63]. Our cytokine array screen also identified TGF-β1 as upregulated in hypoxic astrocytes. Increased expression of TGF-β1 from reactive astrocytes has previously been associated with increased invasion of glioma cells [46], as well as increased proliferation of brain–metastatic breast cancer cells in vitro [64].

It has become increasingly clear that most glioma cells exhibit high phenotypic plasticity and can adopt stemness features depending on experimental conditions or microenvironmental cues [65,66,67]. Here, we report an increase in drug efflux capacity (one measure of stemness) of glioma cells cultured on extracellular matrix produced by astrocytes exposed to severe hypoxia. It is yet unclear whether the glioma cell phenotype induced after culture on matrix from hypoxic astrocytes more generally represents increased stemness, as other direct measures such as self-renewal and stem-cell marker expression were beyond the scope of the present investigation. We found that astrocytes produce high levels of VEGF in response to hypoxia. Previous work has shown that VEGFR2, the receptor for VEGF, is expressed in glioma stem-like cells and the interaction between VEGF and VEGFR2 leads to maintenance of the glioma stem-like cell population [44]. Interestingly, there are reports of extracellular matrix-anchored VEGF, along with other growth factors [68,69]. We previously identified soluble Delta Like Non-Canonical Notch Ligand 1 (DLK1) as a factor that promotes glioma growth and stemness and is secreted in the medium of hypoxic astrocytes [19]. Together, these findings suggest that hypoxic astrocytes could be involved in maintaining the stem-like cell phenotype of glioma cells by secreting soluble factors or remodeling the extracellular matrix.

Recently, we have shown that astrocytes respond to irradiation by becoming reactive; they secrete transglutaminase 2 into the extracellular matrix and thus increase the stemness and radiation resistance of glioma cells [33]. Here, we show that temozolomide treatment, as well as intermediate and severe hypoxia, also induce the reactivity of astrocytes. This suggests that astrocytes present in glioma lesions respond to GBM treatments, including radiation and chemotherapy, as well as to intrinsic hallmarks of GBM biology, such as hypoxia, by actively remodeling the tumor microenvironment. These changes affect properties of glioma cells, such as proliferation and stemness, that are associated with worse patient outcome or tumor recurrence [3,33,70] and implicate astrocytes in tumor maintenance and even therapy resistance.

## 5. Conclusions

Astrocytes become reactive in response to hypoxia and extracellular matrix produced by hypoxic astrocytes increased the proliferation and drug efflux ability of glioma cells, indicating that factors produced by hypoxic astrocytes could be potential therapeutic targets in glioblastoma. Further studies are warranted to identify the mechanisms behind this interaction between hypoxic astrocytes and glioma cells.

## Figures and Tables

**Table 1:** Primer Sequences for RT-qPCR.

Gene	Forward primer (5′-3′)	Reverse primer (5′-3′)
*SDHA*	TGGGAACAAGAGGGCATCTG	CCACCACTGCATCAAATTCATG
*UBC*	ATTTGGGTCGCGGTTCTT	TGCCTTGACATTCTCGATGGT
*YWHAZ*	ACTTTTGGTACATTGTGGCTTCAA	CCGCCAGGACAAACCAGTAT
*MXI1*	AGAGGAGATTGAAGTGGATG	CTGGGTTCTATGAAGTGAATG
*PGK1*	AGATTCAGCTAGTGGCCAAGAGAT	TGCAGTGAAGATGAGCTGAGATG
*BNIP3*	AAAATATTCCCCCCAAGGAGTTC	ACGCTCGTGTTCCTCATGCT
*SLC2A1*	CTTCTATCCCAGGAGGTG	AATGGAGCCTGACCCCTA
*BHLHE40*	CAGTGGCTATGGAGGAGAATCG	GCGTCCGTGGTCACTTTTG
*VEGFA*	CGAAGTGGTGAAGTTCATGGATG	TTCTGTATCAGTCTTTCCTGGTGAG
*CA9*	CCAGGCCTCACTGGCAACT	TCGCCCAGTGGGTCATCT

## Supplementary Materials

The following are available online at https://www.mdpi.com/2073-4409/10/3/613/s1, Figure S1: Astrocytes stabilize HIF-2α more efficiently compared to HIF-1α after exposure to severe hypoxia, Figure S2: Sample gating strategy for the side population assay, Table S1: Upregulated proteins detected in astrocyte lysates from 3 independent donors after exposure to hypoxia.

## References

[1] Stupp R., Hegi M.E., Mason W.P., Bent M.J.V.D., Taphoorn M.J.B., Janzer R.C., Ludwin S.K., Allgeier A., Fisher B., Belanger K. (2009). Effects of radiotherapy with concomitant and adjuvant temozolomide versus radiotherapy alone on survival in glioblastoma in a randomised phase III study: 5-year analysis of the EORTC-NCIC trial. Lancet Oncol..

[2] Huse J.T., Holland E.C. (2010). Targeting brain cancer: Advances in the molecular pathology of malignant glioma and medulloblastoma. Nat. Rev. Cancer.

[3] Lathia J.D., Mack S.C., Mulkearns-Hubert E., Valentim C.L., Rich J.N. (2015). Cancer stem cells in glioblastoma. Genes Dev..

[4] Li Z., Bao S., Wu Q., Wang H., Eyler C., Sathornsumetee S., Shi Q., Cao Y., Lathia J., McLendon R.E. (2009). Hypoxia-Inducible Factors Regulate Tumorigenic Capacity of Glioma Stem Cells. Cancer Cell.

[5] Hambardzumyan D., Bergers G. (2015). Glioblastoma: Defining Tumor Niches. Trends Cancer.

[6] Semenza G.L. (2012). Hypoxia-inducible factors: Mediators of cancer progression and targets for cancer therapy. Trends Pharmacol. Sci..

[7] Duan C. (2016). Hypoxia-inducible factor 3 biology: Complexities and emerging themes. Am. J. Physiol. Cell.

[8] Ivan M., Kaelin W.G. (2017). The EGLN-HIF O_2_-Sensing System: Multiple Inputs and Feedbacks. Mol. Cell.

[9] Koh M.Y., Powis G. (2009). HAF: The new player in oxygen-independent HIF-1α degradation. Cell Cycle.

[10] Li Z., Rich J.N. (2010). Hypoxia and Hypoxia Inducible Factors in Cancer Stem Cell Maintenance. Curr. Top. Microbiol. Immunol..

[11] Heddleston J.M., Li Z., Lathia J.D., Bao S., Hjelmeland A.B., Rich J.N. (2010). Hypoxia inducible factors in cancer stem cells. Br. J. Cancer.

[12] Kaur B., Khwaja F.W., Severson E.A., Matheny S.L., Brat D.J., Van Meir E.G. (2005). Hypoxia and the hypoxia-inducible-factor pathway in glioma growth and angiogenesis. Neuro Oncol..

[13] Harada H. (2016). Hypoxia-inducible factor 1–mediated characteristic features of cancer cells for tumor radioresistance. J. Radiat. Res..

[14] Tang L., Wei F., Wu Y., He Y., Shi L., Xiong F., Gong Z., Guo C., Li X., Deng H. (2018). Role of metabolism in cancer cell radioresistance and radiosensitization methods. J. Exp. Clin. Cancer Res..

[15] Al Tameemi W., Dale T.P., Al-Jumaily R.M.K., Forsyth N.R. (2019). Hypoxia-Modified Cancer Cell Metabolism. Front. Cell Dev. Biol..

[16] Brahimi-Horn M.C., Pouysségur J. (2007). Hypoxia in cancer cell metabolism and pH regulation. Essays Biochem..

[17] Johansson E., Grassi E.S., Pantazopoulou V., Tong B., Lindgren D., Berg T.J., Pietras E.J., Axelson H., Pietras A. (2017). CD44 Interacts with HIF-2α to Modulate the Hypoxic Phenotype of Perinecrotic and Perivascular Glioma Cells. Cell Rep..

[18] Calabrese C., Poppleton H., Kocak M., Hogg T.L., Fuller C., Hamner B., Oh E.Y., Gaber M.W., Finklestein D., Allen M. (2007). A Perivascular Niche for Brain Tumor Stem Cells. Cancer Cell.

[19] Grassi E.S., Jeannot P., Pantazopoulou V., Berg T.J., Pietras A. (2020). Niche-derived soluble DLK1 promotes glioma growth. Neoplasia.

[20] Grassi E.S., Pantazopoulou V., Pietras A. (2020). Hypoxia-induced release, nuclear translocation, and signaling activity of a DLK1 intracellular fragment in glioma. Oncogene.

[21] Tong B., Pantazopoulou V., Johansson E., Pietras A. (2018). The p75 neurotrophin receptor enhances HIF-dependent signaling in glioma. Exp. Cell Res..

[22] Quail D.F., Joyce J.A. (2017). The Microenvironmental Landscape of Brain Tumors. Cancer Cell.

[23] Mega A., Nilsen M.H., Leiss L.W., Tobin N.P., Miletic H., Sleire L., Strell C., Nelander S., Krona C., Hägerstrand D. (2020). Astrocytes enhance glioblastoma growth. Glia.

[24] Sin W.C., Aftab Q., Bechberger J.F., Leung J.H., Chen H., Naus C.C. (2016). Astrocytes promote glioma invasion via the gap junction protein connexin43. Oncogene.

[25] Oushy S., Hellwinkel J.E., Wang M., Nguyen G.J., Gunaydin D., Harland T.A., Anchordoquy T.J., Graner M.W. (2017). Glioblastoma multiforme-derived extracellular vesicles drive normal astrocytes towards a tumour-enhancing phenotype. Philos. Trans. R. Soc. B Biol. Sci..

[26] Herrera-Perez M., Voytik-Harbin S.L., Rickus J.L. (2015). Extracellular Matrix Properties Regulate the Migratory Response of Glioblastoma Stem Cells in Three-Dimensional Culture. Tissue Eng. Part A.

[27] Rath B.H., Fair J.M., Jamal M., Camphausen K., Tofilon P.J. (2013). Astrocytes Enhance the Invasion Potential of Glioblastoma Stem-Like Cells. PLoS ONE.

[28] Kim S.-J., Kim J.-S., Park E.S., Lee J.-S., Lin Q., Langley R.R., Maya M., He J., Kim S.-W., Weihua Z. (2011). Astrocytes Upregulate Survival Genes in Tumor Cells and Induce Protection from Chemotherapy. Neoplasia.

[29] Chen W., Wang N., Du X., He Y., Chen S., Shao Q., Ma C., Huang B., Chen A., Zhao P. (2015). Glioma cells escaped from cytotoxicity of temozolomide and vincristine by communicating with human astrocytes. Med Oncol..

[30] Lin Q., Balasubramanian K., Fan D., Kim S.-J., Guo L., Wang H., Bar-Eli M., Aldape K.D., Fidler I.J. (2010). Reactive Astrocytes Protect Melanoma Cells from Chemotherapy by Sequestering Intracellular Calcium through Gap Junction Communication Channels. Neoplasia.

[31] Priego N., Valiente M. (2019). The Potential of Astrocytes as Immune Modulators in Brain Tumors. Front. Immunol..

[32] Sofroniew M.V., Vinters H.V. (2010). Astrocytes: Biology and pathology. Acta Neuropathol..

[33] Berg T.J., Marques C., Pantazopoulou V., Johansson E., Von Stedingk K., Lindgren D., Jeannot P., Pietras E.J., Bergstrom T., Swartling F.J. (2021). The irradiated brain microenvironment supports glioma stemness and survival via astrocyte-derived Transglutaminase 2. Cancer Res..

[34] Holland E.C., Hively W.P., Depinho R.A., Varmus H.E. (1998). A constitutively active epidermal growth factor receptor cooperates with disruption of G1 cell-cycle arrest pathways to induce glioma-like lesions in mice. Genes Dev..

[35] Ozawa T., Riester M., Cheng Y.-K., Huse J.T., Squatrito M., Helmy K., Charles N., Michor F., Holland E.C. (2014). Most Human Non-GCIMP Glioblastoma Subtypes Evolve from a Common Proneural-like Precursor Glioma. Cancer Cell.

[36] Pietras A., Katz A.M., Ekström E.J., Wee B., Halliday J.J., Pitter K.L., Werbeck J.L., Amankulor N.M., Huse J.T., Holland E.C. (2014). Osteopontin-CD44 Signaling in the Glioma Perivascular Niche Enhances Cancer Stem Cell Phenotypes and Promotes Aggressive Tumor Growth. Cell Stem Cell.

[37] McQuin C., Goodman A., Chernyshev V., Kamentsky L., Cimini B.A., Karhohs K.W., Doan M., Ding L., Rafelski S.M., Thirstrup D. (2018). CellProfiler 3.0: Next-generation image processing for biology. PLoS Biol..

[38] Schindelin J., Arganda-Carreras I., Frise E., Kaynig V., Longair M., Pietzsch T., Preibisch S., Rueden C., Saalfeld S., Schmid B. (2012). Fiji: An open-source platform for biological-image analysis. Nat. Methods.

[39] Vandesompele J., De Preter K., Pattyn F., Poppe B., Van Roy N., De Paepe A., Speleman F. (2002). Accurate normalization of real-time quantitative RT-PCR data by geometric averaging of multiple internal control genes. Genome Biol..

[40] Bleau A.-M., Hambardzumyan D., Ozawa T., Fomchenko E.I., Huse J.T., Brennan C.W., Holland E.C. (2009). PTEN/PI3K/Akt Pathway Regulates the Side Population Phenotype and ABCG2 Activity in Glioma Tumor Stem-like Cells. Cell Stem Cell.

[41] Sofroniew M.V. (2009). Molecular dissection of reactive astrogliosis and glial scar formation. Trends Neurosci..

[42] McKeown S.R. (2014). Defining normoxia, physoxia and hypoxia in tumours—implications for treatment response. Br. J. Radiol..

[43] Keeley T.P., Mann G.E. (2019). Defining Physiological Normoxia for Improved Translation of Cell Physiology to Animal Models and Humans. Physiol. Rev..

[44] Hamerlik P., Lathia J.D., Rasmussen R., Wu Q., Bartkova J., Lee M., Moudry P., Bartek J., Fischer W., Lukas J. (2012). Autocrine VEGF–VEGFR2–Neuropilin-1 signaling promotes glioma stem-like cell viability and tumor growth. J. Exp. Med..

[45] Tarassishin L., Lim J., Weatherly D.B., Angeletti R.H., Lee S.C. (2014). Interleukin-1-induced changes in the glioblastoma secretome suggest its role in tumor progression. J. Proteom..

[46] Kim J.-K., Jin X., Sohn Y.-W., Jin X., Jeon H.-Y., Kim E.-J., Ham S.W., Jeon H.-M., Chang S.-Y., Oh S.-Y. (2014). Tumoral RANKL activates astrocytes that promote glioma cell invasion through cytokine signaling. Cancer Lett..

[47] Xia W., Fu W., Cai X., Wang M., Chen H., Xing W., Wang Y., Zou M., Xu T., Xu N. (2015). Angiogenin Promotes U87MG Cell Proliferation by Activating NF-κB Signaling Pathway and Downregulating Its Binding Partner FHL3. PLoS ONE.

[48] Lee J.W., Chung H.Y., Ehrlich L.A., Jelinek D.F., Callander N.S., Roodman G.D., Choi S.J. (2004). IL-3 expression by myeloma cells increases both osteoclast formation and growth of myeloma cells. Blood.

[49] Puchalski R.B., Shah N., Miller J., Dalley R., Nomura S.R., Yoon J.-G., Smith K.A., Lankerovich M., Bertagnolli D., Bickley K. (2018). An anatomic transcriptional atlas of human glioblastoma. Science.

[50] Wee B., Pietras A., Ozawa T., Bazzoli E., Podlaha O., Antczak C., Westermark B., Nelander S., Uhrbom L., Forsberg-Nilsson K. (2016). ABCG2 regulates self-renewal and stem cell marker expression but not tumorigenicity or radiation resistance of glioma cells. Sci. Rep..

[51] Kucharzewska P., Christianson H.C., Welch J.E., Svensson K.J., Fredlund E., Ringnér M., Mörgelin M., Bourseau-Guilmain E., Bengzon J., Belting M. (2013). Exosomes reflect the hypoxic status of glioma cells and mediate hypoxia-dependent activation of vascular cells during tumor development. Proc. Natl. Acad. Sci. USA.

[52] Wang X., Li C., Chen Y., Hao Y., Zhou W., Chen C., Yu Z. (2008). Hypoxia enhances CXCR4 expression favoring microglia migration via HIF-1α activation. Biochem. Biophys. Res. Commun..

[53] Choudhury G.R., Ding S. (2016). Reactive astrocytes and therapeutic potential in focal ischemic stroke. Neurobiol. Dis..

[54] Sims N.R., Yew W.P. (2017). Reactive astrogliosis in stroke: Contributions of astrocytes to recovery of neurological function. Neurochem. Int..

[55] Holmquist-Mengelbier L., Fredlund E., Löfstedt T., Noguera R., Navarro S., Nilsson H., Pietras A., Vallon-Christersson J., Borg, Åke, Gradin K. (2006). Recruitment of HIF-1α and HIF-2α to common target genes is differentially regulated in neuroblastoma: HIF-2α promotes an aggressive phenotype. Cancer Cell.

[56] Löfstedt T., Fredlund E., Holmquist-Mengelbier L., Pietras A., Ovenberger M., Poellinger L., Påhlman S. (2007). Hypoxia Inducible Factor-2α in Cancer. Cell Cycle.

[57] Brandao M., Simon T., Critchley G., Giamas G. (2019). Astrocytes, the rising stars of the glioblastoma microenvironment. Glia.

[58] Mense S.M., Sengupta A., Zhou M., Lan C., Bentsman G., Volsky D.J., Zhang L. (2006). Gene expression profiling reveals the profound upregulation of hypoxia-responsive genes in primary human astrocytes. Physiol. Genom..

[59] Yoshioka N., Wang L., Kishimoto K., Tsuji T., Hu G.-F. (2006). A therapeutic target for prostate cancer based on angiogenin-stimulated angiogenesis and cancer cell proliferation. Proc. Natl. Acad. Sci. USA.

[60] Zhang H., Gao X., Weng C., Xu Z. (2008). Interaction between angiogenin and fibulin 1: Evidence and implication. Acta Biochim. et Biophys. Sin..

[61] Hong X., Jiang F., Kalkanis S.N., Zhang Z.G., Zhang X.-P., Decarvalho A.C., Katakowski M., Bobbitt K., Mikkelsen T., Chopp M. (2006). SDF-1 and CXCR4 are up-regulated by VEGF and contribute to glioma cell invasion. Cancer Lett..

[62] Zagzag D., Lukyanov Y., Lan L., Ali M.A., Esencay M., Mendez O., Yee H., Voura E.B., Newcomb E.W. (2006). Hypoxia-inducible factor 1 and VEGF upregulate CXCR4 in glioblastoma: Implications for angiogenesis and glioma cell invasion. Lab. Investig..

[63] Jin P., Shin S.-H., Chun Y.-S., Shin H.-W., Shin Y.J., Lee Y., Kim D., Nam D.-H., Park J.-W. (2018). Astrocyte-derived CCL20 reinforces HIF-1-mediated hypoxic responses in glioblastoma by stimulating the CCR6-NF-κB signaling pathway. Oncogene.

[64] Sierra A., E Price J., García-Ramirez M., Méndez O., López L., Fabra A. (1997). Astrocyte-derived cytokines contribute to the metastatic brain specificity of breast cancer cells. Lab. Investig..

[65] Batlle E., Clevers H. (2017). Cancer stem cells revisited. Nat. Med..

[66] Dirkse A., Golebiewska A., Buder T., Nazarov P.V., Muller A., Poovathingal S., Brons N.H.C., Leite S., Sauvageot N., Sarkisjan D. (2019). Stem cell-associated heterogeneity in Glioblastoma results from intrinsic tumor plasticity shaped by the microenvironment. Nat. Commun..

[67] Mitchell K., Troike K., Silver D.J., Lathia J.D. (2021). The evolution of the cancer stem cell state in glioblastoma: Emerging insights into the next generation of functional interactions. Neuro Oncol..

[68] Chen T.T., Luque A., Lee S., Anderson S.M., Segura T., Iruela-Arispe M.L. (2010). Anchorage of VEGF to the extracellular matrix conveys differential signaling responses to endothelial cells. J. Cell Biol..

[69] Taipale J., Keski-Oja J. (1997). Growth factors in the extracellular matrix. FASEB J..

[70] Bao S., Wu Q., McLendon R.E., Hao Y., Shi Q., Hjelmeland A.B., Dewhirst M.W., Bigner D.D., Rich J.N. (2006). Glioma stem cells promote radioresistance by preferential activation of the DNA damage response. Nature.

