# Supplementary material for: Hypoxia-Induced Reactivity of Tumor-Associated Astrocytes Affects Glioma Cell Properties
Source: Cells. 2021 Mar 10;10(3):613. doi: 10.3390/cells10030613 (PMC7999295; doi:10.3390/cells10030613)
Supplement: Supplementary file 1 [file cells-10-00613-s001.pdf]

Figure S1

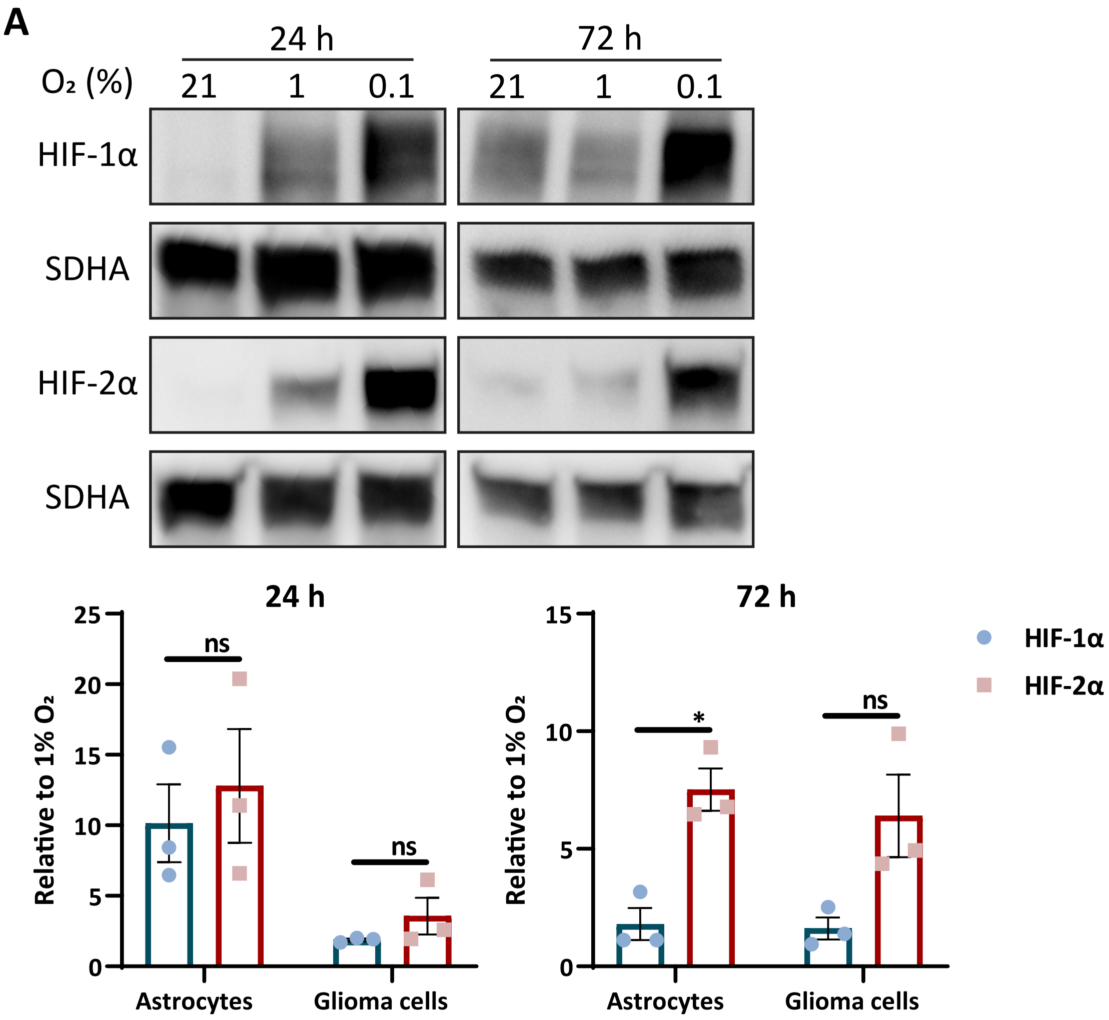

Figure S1. Astrocytes stabilize HIF-2α more efficiently compared to HIF-1α after exposure to extreme hypoxia

(A) Representative images of western blots showing HIF-1α, HIF-2α, and SDHA (loading control) expression in U251MG cells cultured for 24 or 72 h at 21%, 1% or 0.1% O_2. The graphs show quantification of HIF-1α and HIF-2α signal in astrocytes (Figure 1A) and U251MG cells cultured at 0.1% O_2 relative to 1% O_2. *$p < 0.05$; ns, non-significant, two-way ANOVA (Tukey's multiple comparisons test). Data represent mean ± SEM from three independent experiments.

Figure S2

A

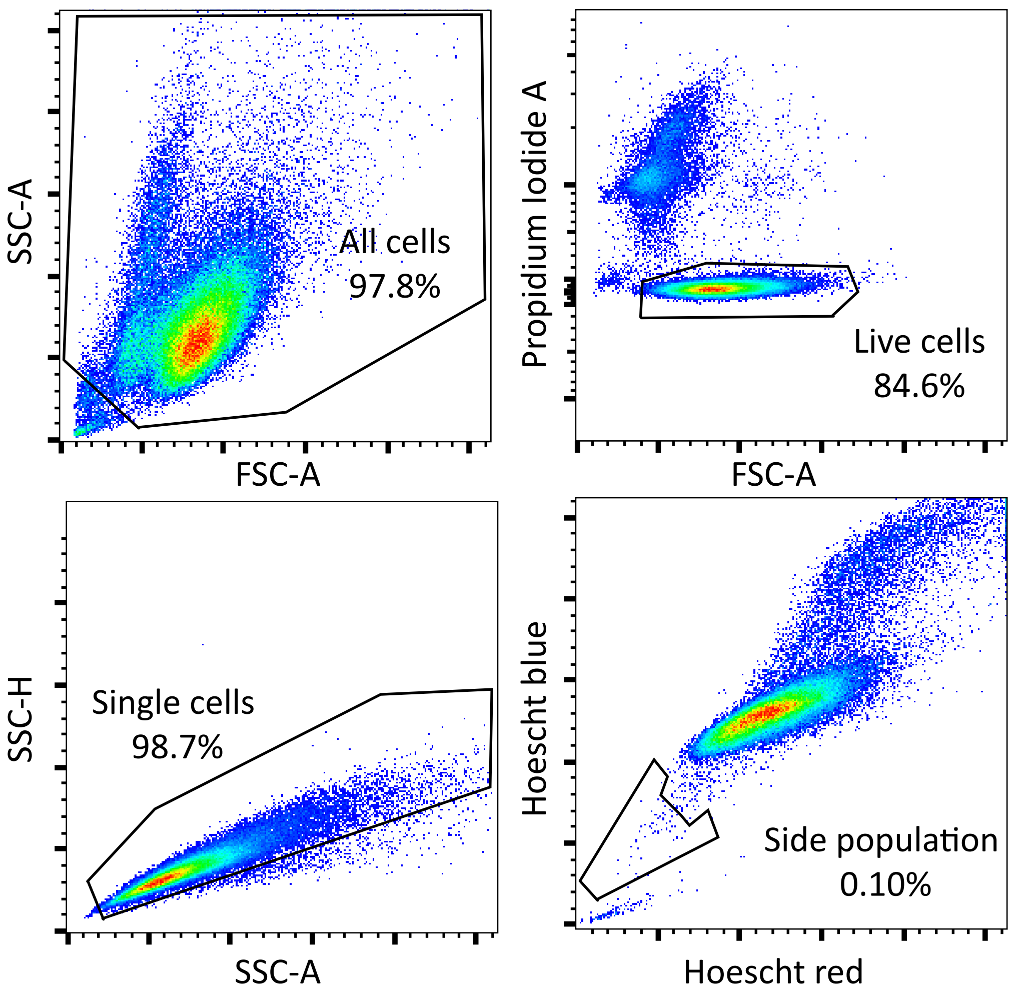

Figure S2. Sample gating strategy for the side population assay.

Sample gating strategy for FTC control for the side population assay.

Table S1

O_2 (%)	1				0.1			
**Astrocyte donor**	**1**	**2**	**3**	**3**	**1**	**2**	**3**	**3**
	IL-1 alpha	IL-1 alpha	GM-CSF	MIG	IL-2	IL-2	**VEGF**	MIG
	IL-13	IL-4	IL-15		**Angiogenin**	**Angiogenin**	IL-15	IL-13
	MCP-2	IL-10	TNF-β		**VEGF**	IL-10	IFN-γ	**IL-1 alpha**
	IL-4	**TGF-β1**	IFN-γ		IL-4	I-309	TNF-β	**Angiogenin**
	EGF	GRO-α	GRO-α		SDF-1	GRO-α	IL-8	**VEGF**
	Angiogenin	I-309	MCP-1		**IL-1 alpha**	**IL-1 alpha**	SDF-1	GM-CSF
	**IL-3**	**IL-3**	IL-10		IL-12 p40/p70	EGF	IL-1β	IL-15
	SDF-1		**TGF-β1**		IL-10	TGF-β1	TNF-α	IL-8
	**TGF-β1**		MCSF			IL-3	MIP-1δ	IGF-I
	IFN-γ		MIP-1δ			SCF	IL-10	PDGF BB
	IL-1β		TNF-α			IL-6	I-309	IL-5
	MCSF		ENA-78			**VEGF**	IL-5	MIP-1δ
	VEGF		**IL-3**			IL-15	EGF	
	IL-5		Leptin			MDC	ENA-78	
			SDF-1			IL-4	MCSF	
			MCP-2			TARC	**Angiogenin**	
			IL-5			IGF-I	MCP-3	
			Oncostatin M			Leptin	GRO-α	
			IL-12 p40/p70			RANTES	Leptin	
			TARC			Oncostatin M	Oncostatin M	
			RANTES				GRO	
			IL-8				MCP-1	
			VEGF				**IL-1 alpha**	
			GRO				IL-2	
							IL-4	
							GM-CSF	
							IL-12 p40/p70	
							TARC	
							IL-6	
							MCP-2	
							TGF-β1	
							SCF	
							RANTES	
							MDC	
							IGF-I	

Table S1: Upregulated proteins detected in astrocyte lysates from 3 independent donors after exposure to hypoxia
